# Cerebral venous thrombosis and myeloproliferative neoplasms: A three‐center study of 74 consecutive cases

**DOI:** 10.1002/ajh.26336

**Published:** 2021-09-10

**Authors:** Naseema Gangat, Paola Guglielmelli, Silvia Betti, Faiqa Farrukh, Alessandra Carobbio, Tiziano Barbui, Alessandro M. Vannucchi, Valerio De Stefano, Ayalew Tefferi

**Affiliations:** ^1^ Division of Hematology Mayo Clinic Rochester Minnesota USA; ^2^ Department of Experimental and Clinical Medicine, CRIMM, Center Research and Innovation of Myeloproliferative Neoplasms, Azienda Ospedaliera Universitaria Careggi University of Florence Florence Italy; ^3^ Section of Hematology, Department of Radiological and Hematological Sciences Catholic University, Fondazione Policlinico A. Gemelli IRCCS Rome Italy; ^4^ Foundation of Clinical Research‐FROM Ospedale Papa Giovanni XXIII Bergamo Italy

## Abstract

The recent association of cerebral venous thrombosis (CVT) with COVID‐19 vaccinations prompted the current retrospective review of 74 cases of CVT (median age = 44 years, range 15–85; 61% females) associated with myeloproliferative neoplasms (MPNs), seen at the Mayo Clinic, Catholic University of Rome, and University of Florence, between 1991 and 2021. Disease‐specific frequencies were 1.3% (39/2893), 1.2% (21/1811) and 0.2% (3/1888) for essential thrombocythemia, polycythemia vera and primary myelofibrosis, respectively. Cerebral venous thrombosis occurred either prior to (*n* = 20, 27%), at (*n* = 32, 44%) or after (*n* = 22) MPN diagnosis. A total of 72% of patients presented with headaches. Transverse (51%), sagittal (43%) and sigmoid sinuses (35%) were involved with central nervous system hemorrhage noted in 10 (14%) patients. In all, 91% of tested patients harbored *JAK2V617F*. An underlying thrombophilic condition was identified in 19 (31%) cases and history of thrombosis in 10 (14%). Treatment for CVT included systemic anticoagulation alone (*n* = 27) or in conjunction with aspirin (*n* = 24), cytoreductive therapy (*n* = 14), or both (*n* = *9*). At a median follow‐up of 5.1 years (range 0.1–28.6), recurrent CVT was documented in three (4%) patients while recurrent arterial and venous thromboses and major hemorrhage were recorded in 11%, 9% and 14%, respectively. Follow‐up neurological assessment revealed headaches (*n* = 9), vision loss (*n* = 1) and cognitive impairment (*n* = 1). The current study lends clarity to MPN‐associated CVT and highlights its close association with *JAK2*V617F, younger age and female gender. Clinical features that distinguish COVID vaccine‐related CVT from MPN‐associated CVT include, in the latter, lower likelihood of concurrent venous thromboses and intracerebral hemorrhage; as a result, MPN‐associated CVT was not fatal.

## 1 INTRODUCTION

Cerebral venous thrombosis (CVT) is a rare event, and the incidence in the general population is estimated at 15.7 cases per million per year.^1^ Cerebral venous thrombosis occurs in < 1% of MPN patients,^2^ but overall an overt MPN is the underlying disease in 3.8% of CVT patients,^3^ suggesting a link between the two conditions. Additionally, *JAK2*V617F mutation was present in 4.8% and 6.6% of CVT patients with or without evidence of an overt MPN, respectively.^4^, ^5^, ^6^ In a European Leukemia Net (ELN) study which included 48 MPN patients with CVT, prominent associations with CVT included the presence of an underlying thrombophilia and *JAK2*V617F mutation in patients with essential thrombocythemia (ET).^2^ Notably, in that particular study, a two‐fold increased risk of recurrent thrombosis was observed among MPN patients with CVT versus other venous thromboses, despite utilization of long‐term antithrombotic therapy in CVT cases.^2^ Accordingly, predictors of recurrent thrombosis and effective therapies to mitigate the excessive risk associated with such events remain to be determined.

The recent association of CVT with COVID‐19 vaccinations^7^, ^8^, ^9^, ^10^ motivated the current review. Our primary objectives were: (1) to provide an estimation of the incidence of CVT in the context of MPN subtypes, followed by a description of clinical phenotype and therapeutic strategies; (2) to determine the long‐term outlook of MPN patients with CVT in terms of recurrent thrombosis, major hemorrhagic events, neurological sequelae and survival; and (3) to identify salient clinical and laboratory features that distinguish MPN‐associated CVT from COVID vaccine‐related CVT.

## 2 METHODS

The current retrospective three‐center series was conducted following institutional review board approval and included a total of 74 consecutive MPN patients with CVT who underwent evaluation at the Mayo Clinic, Rochester MN, USA (*n* = 36), Catholic University of Rome, Italy, (*n* = 23), and University of Florence, Florence, Italy (*n* = 15), between 1991 and 2021. Additionally, the cohort from a previously published multi‐center study that included 42 MPN patients with CVT, who were not included in the current study, was used for comparison of observations. Diagnosis of CVT was established with computed tomography or magnetic resonance imaging (MRI) with venography and all events that occurred either prior to, at or after diagnosis of MPN were included. Classification of MPNs was according to the World Health Organization (WHO) 2016 criteria.^11^ Comprehensive thrombophilia testing was performed in most patients, which included evaluation for factor V Leiden, prothrombin G2010A mutations, protein C, S, anti‐thrombin deficiency, anti‐phospholipid antibody syndrome and paroxysmal nocturnal hemoglobinuria. Major arterial and venous thrombotic events included myocardial infarction, angina, ischemic stroke, transient ischemic attack, peripheral arterial thrombosis, deep venous thrombosis, pulmonary embolism and splanchnic venous events. Major hemorrhage comprised bleeding events that required red cell transfusions, resulted in 2 g/dl decline in hemoglobin or involved critical organs. All clinical and laboratory parameters are summarized as frequency (%) for categorical variables, and median (range) for continuous variables. Comparison between categorical variables was performed by chi‐squared test and continuous variables by Wilcoxon/Kruskal–Wallis tests. A value of *p* ≤ 0.05 was considered significant. The JMP Pro 14.0.0 software package, SAS Institute, Cary, NC was utilized for all analyses.

## 3 RESULTS

### 3.1 Patient characteristics at time of CVT

In all, 74 patients with CVT (median age 44 years, range 15–85; 61% females) that was associated with MPN were evaluated; disease‐specific frequencies were 1.3% (39/2893), 1.2% (21/1811) and 0.2% (3/1888) for ET, polycythemia vera (PV) and primary myelofibrosis (PMF), respectively. Table 1 outlines associated presenting features including details on management and long‐term outlook for MPN patients with CVT. Cerebral venous thrombosis occurred either prior to (*n* = 20, 27%, median time to MPN diagnosis = 16.5 months), at (*n* = 32, 44%) or after (*n* = 22, 30%, median time to CVT = 26 months) MPN diagnosis. A total of 72% of patients presented with headaches, 22% with visual changes, 12% with nausea/vomiting, 8% with neurological deficits, and 6% with seizures. Transverse (51%), sagittal (43%), sigmoid (35%), dural venous/cortical vein (15%), cavernous sinus (14%) and internal jugular veins (14%) were involved with concomitant central nervous system (CNS) hemorrhage, noted in 10 (14%) patients.

**TABLE 1:** Clinical and laboratory characteristics at presentation and outcomes of 74 patients with cerebral venous thrombosis (CVT) and myeloproliferative neoplasm (MPN)

Variables at presentation	Cerebral venous thrombosis		Myeloproliferative neoplasm
Age (years) (median [range])	44 (15–85)		44 (13–84.5)
Female gender (*n* [%])	45 (61)		
Hemoglobin (g/dL) (median [range])	14.7 (7.7–20)		14.5 (9.5–20)
Leukocyte count (× 10^9^/L) (median [range])	10.2 (4.6–30.9)		9.5 (3.9–25.6)
Platelet count (× 10^9^/L) (median [range])	536 (134–2000)		553 (100–2000)
Cardiovascular risk factors (*n* [%])		MPN type (*n* [%])	
Diabetes mellitus	4 (5)	ET	39 (53)
Hypertension	19 (26)	PV	21 (28)
Smoking	10 (14)	Pre‐PMF	6 (8)
		MPN‐U	4 (5)
		PMF	3 (4)
		Post‐PV MF	1 (1)
Thrombophilia^a^ including any other predisposition (*n* [%])	*n* = 61	Driver mutation (*n* [%]):	*n* = 65
None	42 (69)		
Antiphospholipid antibody	6 (10)	*JAK2V617F*	59 (91)
Prothrombin G20210A (HET)	6 (10)	*CALR*	2 (3)
Factor V Leiden (HET)	2 (3)	*Type 1 CALR*	2(3)
Protein S deficiency	1 (2)	*Type 2 CALR*	0 (0)
Hyperhomocysteinemia	3 (5)	*MPL*	1 (2)
Oral contraceptive	2 (3)	Triple negative	3 (5)
Neurosurgery/mastoiditis	3 (5)		
Prior thrombosis^b^ (*n* [%])	10 (14)		17 (23)
Major arterial thrombosis	5 (7)		6 (8)
Major venous thrombosis excluding CVT	5(7)		11 (15)
Timing of CVT in relation to MPN (*n* [%]) (median/range [in months])	*n* = 73		
Prior to diagnosis of MPN	20 (27)		
	(16.5/1–96)		
At MPN diagnosis	32 (44)		
After diagnosis of MPN	22 (30)		
	(26/1–251)		
Presenting symptoms^c^ (*n* [%])	*n* = 50		
Headache	36 (72)		
Visual changes	11(22)		
Nausea/vomiting	6(12)		
Location^d^ (*n* [%])			
Transverse sinus	38 (51)		
Sagittal sinus	32 (43)		
Sigmoid sinus	26 (35)		
Dural venous/cortical vein	11 (15)		
Cavernous sinus	10 (14)		
Internal jugular vein	10 (14)		
Central nervous system hemorrhage (*n* [%])	10 (14)		
Treatment for CVT (*n* [%])		Treatment at time of CVT (*n* [%]):	*n* = 21
Anticoagulation alone	27 (36)	None	8 (38)
Anticoagulation + aspirin	24 (32)	Anticoagulation	5 (24)
Anticoagulation + cytoreductive therapy	14 (19)	Aspirin	5 (24)
Anticoagulation + aspirin + cytoreductive therapy	9 (12)	Cytoreductive therapy	7 (33)
**Outcomes**			
Follow‐up in years (median [range])	5.1 (0.1–28.6)		6.4 (0.1–28.6)
Recurrent CVT (*n* [%])	3 (4)		
Persistent neurological symptoms (*n* [%])	11/30 (37)		
Major arterial thrombosis (*n* [%])	8 (11)		9 (12)
Major venous thrombosis excluding CVT	7 (9)		7 (9)
Major hemorrhage^e^ (*n* [%])	10/73 (14)		10/73 (14)
Fibrotic transformation (*n* [%])	5/64 (8)		5/64 (8)
Leukemic transformation (*n* [%])	1 (1)		1 (1)
Deaths (*n* [%])	5 (7)		5 (7)

Abbreviations: ET, essential thrombocythemia; HET, heterozygous; MPN‐U, myeloproliferative neoplasm‐unclassified; PMF, primary myelofibrosis; post‐PV‐MF, post‐PV myelofibrosis; pre‐PMF, pre‐fibrotic primary myelofibrosis; PV, polycythemia vera.

^a^ Thrombophilia testing included factor V Leiden, prothrombin gene mutation, protein C, S, anti‐phospholipid antibody, paroxysmal nocturnal hemoglobinuria (PNH), and homocysteine levels.

^b^ Major arterial thrombosis included cerebrovascular accident, transient ischemic attack, myocardial infarction, angina, peripheral arterial thrombosis. Major venous thrombosis included deep vein thrombosis, pulmonary embolism, splanchnic vein thrombosis.

^c^ Patients had one or more symptoms; only frequent symptoms (> 10%) have been provided.

^d^ Patients had CVT at one or more location.

^e^ Major hemorrhage consisted of bleeding events that either required red cell transfusions, resulted in 2 g/dL decline in hemoglobin or involved critical organs.

Myeloproliferative neoplasm phenotype included ET (*n* = 39, 53%), PV (*n* = 21, 28%), pre‐fibrotic MF (*n* = 6, 8%), MPN‐unclassified (*n* = 4, 5%), primary MF (*n* = 3, 4%) and post‐PV MF (*n* = 1, 1%). Driver mutation testing was performed in 65 patients: 91% harbored *JAK2*V617F, 3% harbored *CALR* type 1, 2% harbored *MPL* and 5% were triple negative; moreover, *JAK2*V617F was mutated in 27 out of 33 (82%) ET patients. An underlying thrombophilia or predisposing condition was identified in 19 (31%) cases, 10% each with antiphospholipid antibodies and prothrombin G20210A mutation (Table 1). Notably, one patient received the Ad26.COV2.S vaccine, 5 days prior to presenting with CVT, not associated with thrombosis in other sites, thrombocytopenia or platelet factor four antibodies. A history of thrombosis was documented in 10 (14%) patients, which included three splanchnic venous events.

These observations were similar to those noted in our comparative group from a previously published cohort that included 42 MPN patients with CVT.^2^ Table 2 summarizes the clinical and laboratory characteristics of the comparative cohort (median age 51 years, range 16–84; 55% females). Myeloproliferative neoplasm phenotype was predominantly ET (*n* = 25, 60%), followed by PV (*n* = 11, 26%), and PMF (*n* = *5*, 12%) with *JAK2*V617F mutation present in 81% of patients. Both cohorts were similar with respect to age at CVT, gender, MPN subtype, prevalence of *JAK2*V617F mutation, timing of CVT, presenting symptoms and history of thrombosis. Prior thrombosis was documented in eight (19%) patients in the comparative group, which included four splanchnic venous events. The only discrepant observation was the relatively infrequent occurrence of concomitant CNS hemorrhage in the comparative cohort: 2% versus 14% in our study (*p* = 0.04).

**TABLE 2:** Clinical and laboratory characteristics at presentation and outcomes of 42 patients^a^ with cerebral venous thrombosis (CVT) and myeloproliferative neoplasm (MPN)

Variables at presentation	Cerebral venous thrombosis		Myeloproliferative neoplasm
Age in years (median [range])	51 (16–84)		48 (17–84)
Female gender (*n* [%])	23 (55)		
Hemoglobin g/dL (median [range])	13.9 (8.1–20.7)		15 (9.2–24.2)
Leukocyte count (× 10^9^/L) (median [range])	10 (2.3–16.6)		8.7 (3.5–21.8)
Platelet count (× 10^9^/L) (median [range])	452 (10–1059)		594 (123–1200)
Cardiovascular risk factors (*n* [%])		MPN type (*n* [%]):	
Diabetes mellitus	2 (5)	ET	25 (60)
Hypertension	6 (14)	PV	11 (26)
Smoking	7 (17)	PMF	5 (12)
		Post‐ET MF	1 (2)
Underlying thrombophilia (*n* [%])	15/37 (41)	*JAK2V617F mutated*	*n = 37*
			30 (81)
Prior thrombosis^b^ (*n* [%])	8 (19)		
Major arterial thrombosis	5 (12)		
Major venous thrombosis excluding CVT	4 (10)		
Timing of CVT in relation to MPN (*n* [%]) (median/range in months)			
Prior to diagnosis of MPN	16 (38)		
	(6.5/1–352)		
At MPN diagnosis	11 (26)		
After diagnosis of MPN	15 (36)		
	(52/1–205)		
Presenting symptoms^c^ (*n* [%])	*n* = 33		
Headache	23 (70)		
Visual changes	7 (17)		
Vomiting	7(17)		
Hemiparesis	4 (12)		
Aphasia/dysarthria	4 (12)		
Vertigo	4(12)		
Seizures	3(7)		
Location^d^ (*n* [%])			
Sagittal sinus	21 (50)		
Transverse sinus	15 (36)		
Sigmoid sinus	15 (36)		
Straight sinus	6 (14)		
Dural venous/cortical vein	2 (5)		
Cavernous sinus	2 (5)		
Internal jugular vein	2(5)		
Central nervous system hemorrhage (*n* [%])	1 (12)		
Active therapy at time of CVT (*n* [%])			
None	25 (60)		
Anticoagulation	4 (10)		
Aspirin	7 (17)		
Cytoreductive therapy	13 (31)		
Outcomes			
Follow‐up in years (median [range])	4.5 (0.01–34.6)		6.1 (0.5–34.4)
Recurrent CVT (*n* [%])	1 (2)		
Major arterial thrombosis (*n* [%])	2 (5)		
Major venous thrombosis excluding CVT	12 (29)		
Major hemorrhage^e^ (*n* [%])	6 (14)		
Fibrotic transformation (*n* [%])	2 /36 (6)		2/36 (6)
Leukemic transformation (*n* [%])	1 (2)		1 (2)
Deaths (*n* [%])	5 (12)		5 (12)

Abbreviations: ET, essential thrombocythemia; PMF, primary myelofibrosis; post ET‐MF, post‐ET myelofibrosis; PV, polycythemia vera.

^a^ Comparative cohort from a previously published multi‐center study that included 42 MPN cases with CVT, which were not included in the current study (Martinelli et al.^2^)^.^

^b^ Major arterial thrombosis included cerebrovascular accident, transient ischemic attack, myocardial infarction, angina, peripheral arterial thrombosis. Major venous thrombosis included deep vein thrombosis, pulmonary embolism, splanchnic vein thrombosis.

^c^ Patients had one or more symptom, only frequent symptoms (>5%) have been provided.

^d^ Patients had CVT at one or more location.

^e^ Major hemorrhage consisted of bleeding events that either required red cell transfusions, resulted in 2 g/dL decline in hemoglobin or involved critical organs.

Treatment for CVT included systemic anticoagulation alone in 27 (36%) patients or in conjunction with aspirin (*n* = 24, 32%), cytoreductive therapy (*n* = 14, 19%), or both aspirin and cytoreduction (*n* = 9, 12%). Initial systemic anticoagulation consisted of unfractionated or low‐molecular‐weight heparin, followed by transition to warfarin (*n* = 44), direct oral anticoagulants (*n* = 21), enoxaparin (*n* = 8) or fondaparinux (*n* = 1). Of note, 5/21 (24%) patients with CVT post‐MPN diagnosis were already on systemic anticoagulation at the time of the event. Other active therapies at the time of CVT included aspirin in 5/21 (24%) and cytoreductive therapy in 7/21 (33%), while 8/21 (38%) were not receiving any form of therapy.

### 3.2 Outcome following CVT

At a median follow‐up of 5.1 years (range 0.1–28.6), recurrent CVT was documented in only three (4%) patients; by contrast, incidence rates for other arterial and venous thromboses and major hemorrhage were 11% (2/100 patient‐years), 9% (1.9/100 patient‐years) and 14% (3/100 patient‐years), respectively. Antithrombotic secondary prophylaxis was ongoing in 53% of patients with thrombotic recurrences and 80% of those with hemorrhage. Arterial thrombosis included cardiovascular (*n* = 3), cerebrovascular (*n* = 3) and peripheral arterial events (*n* = 2). Splanchnic venous thrombosis comprised three of seven (43%) venous thrombotic events while the remainder included deep venous thromboses (*n* = 3) and pulmonary embolism (*n* = 1). By contrast, the incidence of venous thrombosis was significantly higher in the aforementioned previously published cohort (12 [29%] vs. 7 [9%], *p* = 0.01), with 5/12 (42%) splanchnic venous events. Furthermore, rates of recurrent arterial thrombosis and major hemorrhage were similar in both cohorts (Tables 1 and 2).

Fibrotic and leukemic transformation was recorded in five patients (8%) and one (1%) patient, respectively, with five (7%) deaths documented to date. Fortunately, CVT by itself did not contribute to mortality in any patient; however, follow‐up neurological assessment revealed significant morbidity in 11/30 (37%) evaluable cases, in the form of chronic headaches (*n* = 9), vision loss (*n* = 1) and cognitive impairment (*n* = 1).

## 4 DISCUSSION

This is the largest compilation of patients with CVT in association with MPN. A prior multi‐center ELN study which included 48 patients with CVT and MPN recruited from 11 hematology centers, predated discovery of the *CALR* mutation; moreover, neurological sequelae were not assessed in that particular study.^2^ The current study highlights the close association of MPN‐associated CVT with *JAK2*V617F mutation, younger age, and female gender.^2^ Although the risk of recurrent CVT was low (4%), recurrent arterial and venous thrombosis excluding CVT was reported in 11% and 9% of patients, respectively, despite ongoing anti‐thrombotic therapy in half the cases. The ELN study reported an exceptionally high rate of recurrent non‐CVT thromboses (42%) despite the majority (80%) of patients receiving anti‐thrombotic therapy. A predilection for splanchnic venous thrombosis both prior to and after CVT was appreciated in our series; three out of five (60%) prior, and three out of seven (43%) subsequent venous thromboses involved the splanchnic veins. These findings were consistent with the ELN study in which splanchnic venous thrombosis occurred in 19% with MPN‐associated CVT versus 11% in MPN patients with other venous thromboses.^2^ Taken together, these observations regarding a heightened risk of recurrent thrombosis following CVT, including the propensity for splanchnic venous events, requires confirmation in controlled studies.

In contrast to our findings, a recent report from China demonstrated a remarkably high incidence of CVT in 23 of 91 (25%) patients with ET.^12^ Akin to our findings, CVT was more likely to occur in women (61%) and younger patients (median age 38 years). Prognosis was generally favorable, with no recurrence or bleeding events, save for one demise related to cerebral herniation.^12^

The natural history of CVT has been extensively studied; in an international prospective observational report of 624 patients with CVT, mortality rate was 8.3%, while moderate impairment or severe handicap was observed in 5.1% of patients^13^ The incidence rates of recurrent CVT and non‐CVT thrombosis were 2.2% and 4.3%, respectively.^13^ The particular study identified age above 37 years, male gender, coma, CNS hemorrhage, infection and cancer as predictors of poor outcome following CVT; subsequently the aforementioned variables were utilized to formulate a risk score enabling identification of high‐risk CVT patients.^14^ In another prospective study which included 187 CVT patients, similar rates of recurrent venous thrombosis were observed; furthermore prior venous thrombosis, presence of cancer or hematological malignancy and unknown cause of CVT were independently associated with recurrence.^15^ On the other hand, in a contemporary series of 17 CVT patients with half receiving long‐term anticoagulation, none of the patients experienced recurrent thrombosis or major hemorrhage; moreover one patient developed residual neurological morbidity.^16^

A renewed interest in CVT stems from its recent association with COVID‐19 vaccinations.^7^, ^8^, ^9^, ^10^ Considering CVT preceded MPN diagnosis in 27% of MPN cases and coincided with it in 44% of MPN cases, it is imperative to distinguish MPN‐associated CVT from vaccine‐related CVT. In a series of 12 patients with CVT after receiving Ad26.COV2.S vaccine in the United States,^7^ all were women <60 years of age (similar to MPN‐associated CVT) without underlying thrombophilia (unlike MPN‐associated CVT). All but one patient presented with headache (similar to MPN‐associated CVT). Seven patients had intracerebral hemorrhage (higher than MPN‐associated CVT), and additionally eight patients experienced concomitant non‐CVT venous thrombosis with two splanchnic venous events (higher than MPN‐associated CVT). A hallmark of vaccine‐related CVT was thrombocytopenia and positive heparin‐induced thrombocytopenia (HIT) antibody by enzyme‐linked immunosorbent assay (ELISA) in 11 patients. All patients were hospitalized, with 10 patients in the intensive care unit which culminated in three deaths. By contrast, no CVT‐related deaths were noted among MPN patients. Similar observations were also reported following the ChAdOx1 vaccine; all nine patients with CVT were younger women (median age 36 years; range 22–49 years).^8^ Five out of nine (56%) CVT patients developed additional thromboses, which included splanchnic venous thrombosis in three patients. All patients demonstrated concomitant thrombocytopenia, and HIT antibody ELISA was strongly positive in four patients. Mortality rate was high, with five deaths reported. Another study from Norway described five healthcare workers aged 32–54 years, 80% women who developed CVT after receiving the ChAdOx1 vaccine.^9^ All but one patient presented with headaches, one with concomitant splanchnic venous thrombosis; importantly all patients were thrombocytopenic. Overall, CVT was fatal in three women (60%). In a nationwide series from the UK of 220 patients with definite or probable vaccine‐induced immune thrombocytopenia and thrombosis (VITT), CVT was the most common thrombotic site at presentation (50%), and in 40 of these patients (36%), CVT was complicated by secondary intracranial hemorrhage. Overall mortality was 22% and the occurrence of CVT increased the odds of death 2.7‐fold.^10^ In a multi‐center report from the UK, which included 95 patients with CVT following COVID vaccine, 70 CVT patients with VITT were compared with 25 patients without VITT. The particular study confirmed a higher likelihood of concurrent venous or arterial thrombosis and intracerebral hemorrhage, resulting in higher mortality and disability in patients with VITT‐associated CVT.^17^ Moreover, institution of non‐heparin anticoagulants and intravenous immunoglobin was found to be associated with improved outcomes.^17^ In lieu of these observations, it is important to recognize the salient clinical features that distinguish COVID vaccine‐related and MPN‐associated CVT, which, in the latter, include a lower likelihood of concomitant non‐CVT venous thromboses and intracerebral hemorrhage with the latter.^7^, ^8^, ^9^, ^10^ Although MPN‐ associated CVT was not fatal, our study sheds light on the substantial non‐thrombotic morbidity ensuing from persistent neurological symptoms reported by a third of patients. In a recent ELN study of MPN‐COVID‐19 it was found that venous thrombosis was significantly more frequent in ET (17%) vs 5% in PV and PMF without any CVT events.^18^ Moreover, none of the patients with MPN in the ELN registry have experienced CVT following COVID vaccination (personal communication‐ Tiziano Barbui M.D. [T.B.]).

In summary, the current study provides MPN subtype‐specific incidence of CVT, and confirms its association with younger age, female gender and *JAK2*V617F mutation. Importantly, our observations lay the groundwork for future investigations geared towards identification of risk factors for recurrent non‐CVT thrombosis, including splanchnic venous thrombosis in MPN patients with CVT. Furthermore, controlled studies are needed to determine the optimal therapeutic approach to ameliorate the risk associated with such events.

## Data Availability

Data available upon request by email to the corresponding author.
